# Customisation of an instrument to assess anaesthesiologists' non-technical skills

**DOI:** 10.5116/ijme.54be.8f08

**Published:** 2015-02-22

**Authors:** Rikke M.H.G. Jepsen, Lene Spanager, Helle T. Lyk-Jensen, Peter Dieckmann, Doris Østergaard

**Affiliations:** 1 Danish Institute for Medical Simulation (DIMS), Capital Region of Denmark and University of Copenhagen, Denmark

**Keywords:** anaesthesiology, non-technical skills, training, assessment, operating room

## Abstract

**Objectives:**

The objectives of the study were to identify Danish anaesthesiologists’ non-technical skills and to customise the Scottish-developed Anaesthetists’ Non-Technical Skills instrument for Danish anaesthesiologists.

**Methods:**

Six semi-structured group interviews were conducted with 31 operating room team members: anaesthesiologists, nurse anaesthetists, surgeons, and scrub nurses. Interviews were transcribed verbatim and analysed using directed content analysis. Anaesthesiologists’ non-technical skills were identified, coded, and sorted using the original instrument as a basis. The resulting prototype instrument was discussed with anaesthesiologists from 17 centres to ensure face validity.

**Results:**

Interviews lasted 46–67 minutes. Identified examples of anaesthesiologists’ good or poor non-technical skills fit the four categories in the original instrument: situation awareness; decision making; team working; and task management. Anaesthesiologists’ leadership role in the operating room was emphasised: the original ‘Task Management’ category was named ‘Leadership’. One new element, ‘Demonstrating self-awareness’ was added under the category ‘Situation Awareness’. Compared with the original instrument, half of the behavioural markers were new, which reflected that being aware of and communicating one’s own abilities to the team; working systematically; and speaking up to avoid adverse events were important skills.

**Conclusions:**

The Anaesthetists’ Non-Technical Skills instrument was customised to a Danish setting using the identified non-technical skills for anaesthesiologists and the original instrument as basis. The customised instrument comprises four categories and 16 underpinning elements supported by multiple behavioural markers. Identifying non-technical skills through semi-structured group interviews and analysing them using direct content analysis proved a useful method for customising an assessment instrument to another setting.

## Introduction

Inadequate use of non-technical skills (NTS) is a contributing factor to more than 70% of in-hospital adverse events.^1^ NTS in healthcare can be defined as the cognitive and social skills underpinning medical knowledge and technical skills needed to contribute to safe and efficient performance.^1^ For example, NTS encompass decision making, team working, and leadership, or behaviours not directly related to medical expertise or the use of drugs or equipment.^2^

An observational study found that failures in operating room (OR) communication occur in approximately 30% of all cases. One-third of these cases were found to have a potentially negative effect on patient safety.^3^ NTS failures in the OR have been associated with a higher risk of technical errors.^4^ These examples support the argument that technical skills and a high level of medical knowledge are necessary but not sufficient to maintain a high-level of patient safety. A Delphi-type study identified a number of NTS as essential for anaesthesiologists’ safe performance.^5^ European societies of anaesthesiology and intensive care medicine have issued declarations emphasising the need for training and research in this area to prevent adverse events.^6^^,^^7^ Thus, properly designed and tested instruments and methods for studying and training NTS are needed. 

Training NTS in the form of crisis resource management programs has been introduced in several healthcare specialties.^8^ Individuals may be trained in NTS with positive implications for patient morbidity and mortality.^9^^,^^10^ Instruments have been developed to assess NTS in the OR for individuals and teams. These instruments are frequently behavioural marker systems that guide the user to assess NTS on the basis of observable behaviours.^11^ They provide a common language for talking about NTS, a structure with which to assess behaviours, and have demonstrated value for training and understanding performance.^12^ The Anaesthetists’ Non-Technical Skills (ANTS) system developed in Scotland is such an instrument.^2^ ANTS comprises four categories of NTS with 15 underpinning elements and numerous behavioural markers that are examples of good and poor behaviour. ANTS was empirically derived from substantial research, including a literature review, examination of existing marker systems, cognitive task analysis through interviews with anaesthesiologists, and cross-checking the instrument in the OR during the iterative development process.^2^

Currently, reliable and valid instruments are lacking to facilitate learning and access NTS during anaesthesiology specialist training in Denmark. Although some parts of instruments used to assess NTS might be applicable in different contexts, no exact knowledge exists about how far such generalisation could reach.^12^-^14^ Previous work with behavioural marker systems for surgeons (Non-Technical Skills for Surgeons in Denmark, NOTSSdk)^15^ and nurse anaesthetists (Nurse Anaesthetists’ Non-Technical Skills, N-ANTS)^16^ indicated that differences exists in tasks, responsibilities, and cultures between Denmark and Scotland. Therefore, we expected that customisation was necessary to adapt ANTS to the Danish setting. Activity Theory as defined by Leont’ev is a possible framework for conceptualising similarities and differences in for example, instruments for workplace-based assessments because it offers an approach to the study of activities in context.^17^ Activity Theory provides three levels of conceptualising customisation needs across contexts: activity, action, and operation. Providing anaesthesia, as an ‘activity’ is similar throughout the world because the goal is the same: inducing sleep, relaxing the muscles, and eliminating pain. However, the ‘actions’ related to this activity are influenced by how the work is organised in a healthcare system. For example, different national procedures and organisational differences may exist. The manners in which these actions are carried out with an individual patient depend on the context and are expressed as concrete ‘operations’ in Activity Theory. Operations are the automatized psychomotor steps needed to implement an action. Thus, Activity Theory can be regarded as a lens through which to interpret and describe the results of the customisation of ANTS to ANTSdk.

The research objectives of this paper were to: identify Danish anaesthesiologists’ good and poor non-technical skills; and, customise the assessment instrument ANTS for Danish anaesthesiologists as ANTSdk.

## Methods

### Design

**Figure 1.**
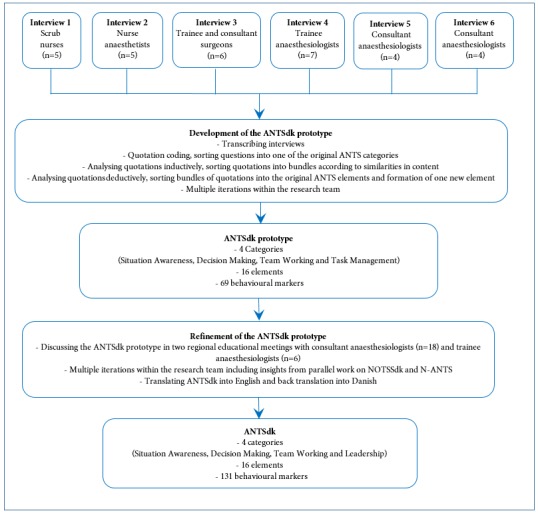
Flowchart illustrating the ANTSdk development process

We conducted this explorative, qualitative study in two steps (Figure 1). We first conducted six semi-structured group interviews with members of the multi-professional OR team to explore their perception of anaesthesiologists’ behaviour. We used directed content analysis of the group interviews to create an ANTSdk prototype,^18^ which was presented to anaesthesiologists at two regional educational council meetings for refinements. The final version of ANTSdk was developed by considering their input. Participants in the semi-structured group interviews and the two regional educational council meetings did not overlap. The study is reported in accordance with the consolidated criteria for reporting qualitative research.^19^ Danish law exempts educational studies from ethical approval because they do not involve biomedical research. An exemption letter from the Regional Ethical Committee of the Capital Region of Denmark was obtained (H-2-2012-FSP16). All participants received written and oral information on the purpose and objectives of the study and informed consent was obtained.

### The Danish anaesthesiology setting

We provide a brief description of the Danish work settings in anaesthesiology to facilitate an understanding of the features of ANTSdk. A team consisting of a consultant anaesthesiologist and a certified registered nurse anaesthetist or a trainee anaesthesiologist usually induces anaesthesia. The nurse anaesthetist or the trainee anaesthesiologist maintains the anaesthesia during the operation. The consultant anaesthesiologist frequently supervises nurses or trainees in more ORs at a time and attends tasks outside the OR.

### Participants

#### Group interviews

Participants in the six semi-structured group interviews comprised 31 anaesthesiologists, surgeons, nurse anaesthetists and scrub nurses who represented multi-professional OR team members from five surgical specialties (orthopaedics, urology, gastrointestinal, mamma, and obstetrics and gynaecology) at a Danish university hospital (Table 1).

**Table 1.** Group interview participants’ demographic data and interviewer information

Interview participants	Number	Gender F/ M	Experience as nurse/physician, median (range) in years	Interviewers^‡^
Scrub nurses	5	5/0	16 (6–31)	HTLJ, RMHGJ
Nurse anaesthetists	5	4/1	20 (7–36)	LS, RMHGJ
Trainee surgeons*	2	0/2	5 (2–8)	LS, RMHGJ
Consultant surgeons*	4	2/2	17,5 (15–20)	LS, RMHGJ
Trainee anaesthesiologists	7	3/4	6 (3–9)	HTLJ, RMHGJ
Consultant anaesthesiologists^†^	8	4/4	21 (13–33)	DO, RMHGJ
Total	31	18/13		

* The trainee and consultant surgeons were interviewed together. †The consultant anaesthesiologists were interviewed in two groups for practical reasons. ‡ HTLJ: nurse anaesthetist and research assistant; RMHGJ: trainee anaesthesiologist and research fellow; LS: trainee surgeon and PhD student; DO: consultant anaesthesiologist and head of the hospital’s simulation centre.

#### Regional educational council meetings

We presented the ANTSdk prototype to 18 consultant anaesthesiologists with educational responsibility and six trainee anaesthesiologists at two regional educational council meetings for refinements to the instrument. These participants represented 17 centres and more than half the educational anaesthesiology departments in Denmark.

### Sampling method

Participants in the group interviews were recruited by asking leaders of the OR staff groups to find volunteers with an interest in discussing anaesthesiologists’ NTS and, thus, represented a convenience sample to fit with the clinical work. Participants in the regional educational council meetings comprised appointed representatives from the departments of anaesthesiology in the specific regions.

### Data collection

#### Group interviews to develop the ANTSdk prototype

We informed participants in the group interviews about the purpose of the study by email before the interviews and orally at the beginning of the interviews. The interviews were performed mono-professionally in a meeting room near the OR and were planned to last one hour. The interview guide was based on ANTS categories and elements, and a pilot interview was conducted to fine-tune the interview guide. We used the same order of asking questions during all interviews. Given the semi-structured manner of the interviews, themes were explored when they emerged. The purpose of the interviews was to facilitate participants’ description of their perceptions of how an anaesthesiologist should or should not perform. Participants were asked to support their statements with examples of anaesthesiologists’ good and poor behaviour. The main author (RMHGJ) and one other member of the research team conducted the interviews that were used to create the ANTSdk prototype (Table 1). The research team encompassed a trainee anaesthesiologist and research fellow (RMHGJ, who was known in the OR from prior employment), a consultant anaesthesiologist and head of the simulation centre located in the hospital (DO), a trainee surgeon and PhD student (LS), a nurse anaesthetist (HTLJ), and a psychologist (PD). The interviews were recorded and transcribed verbatim, and participants’ identities were anonymised.

#### Regional educational council meetings to collect feedback on the ANTSdk prototype

The ANTSdk prototype was then discussed in groups of four to eight participants at the two regional educational council meetings facilitated by two members of the research team. The purposes of these discussions were to discuss issues of understanding, assess face validity, and provide behavioural markers for elements that were not sufficiently supported by behavioural markers from the group interviews.

### Data analysis of group interviews

RMHGJ and HTLJ independently assigned a colour-code in electronic documents to quotations from the group interviews, thus sorting them into one of the four ANTS categories (Situation Awareness, Decision Making, Task Management, and Team Working). They discussed differences in their coding until they reached agreement. RMHGJ created paper copies of the quotations within each category to ensure that each individual quote was on one sheet of paper. Inductively, these quotations were then sorted physically into bundles according to similarities in content. The bundles of quotations were labelled with headings describing their paraphrased contents. Each bundle was then sorted deductively into one of the existing ANTS elements. The bundles that did not fit within the existing elements in the particular category were tested on elements in the other three categories. If a bundle did not fit into the existing elements, it was placed separately. The separate bundles were reviewed for possible new categories or elements at the end of the analysis. RMHGJ made a proposal for one new element on the basis of the bundles that did not fit within the existing elements. RMHGJ re-analysed all the quotations to ensure that they were all in the right place and transformed quotations from the interviews into behavioural markers that matched the elements (Table 2). The sort ing, proposal for a new element, and the behavioural markers were discussed iteratively with the research team, a process that led to the development of the ANTSdk prototype.

**Table 2.** The analytical process from interview quotations to elements and behavioural markers

Quotations	Category	Paraphrase	Element	Behavioural marker
*“Some trainees make you more insecure than others; for example, when they reject a suggestion to call a consultant to attend them with a very ill patient on their first duty.” *NA3	Situation Awareness	Anaesthesiologists’ loss of awareness of own limits has consequences for team members and patients.	Demonstrating self-awareness	Is not aware of when own limits are reached
*“When you are new, you know that there are many things you cannot do. When you are more experienced, you might think you have a lot of experience and it will be alright, that is when you become dangerous.” *TA6				
*“The entire OR team needs to know which decisions are made and the most important reasons for choosing exactly that, so it is understandable to them.” *CA6	Decision making	Importance of providing team members with justification for decisions to help them understand what is going to happen.	Choosing, communicating, and implementing decisions	Justifies and communicates decisions to relevant team members
*“It is possible to briefly justify a decision, especially if you believe that there are more opinions about what is happening right now.” *TA3				
*“I have noticed that communication works better if I know who has which competence and their names. I think the team-making process is very vulnerable, especially in acute situations. If you use names, then you know more where you have each other, it creates more trust.”* CA1	Team working	Importance of presenting oneself to OR team members with name and competence.	Exchanging information	Introduces her/himself to new team members and states competencies
*“I need a doctor or whoever comes through the door to say ‘my name is, I am new, I know this and this, and I am going to be here today,’ then there is already a completely different atmosphere.” *SN3				
*“It is just that the anaesthesiologist needs to orientate himself or herself about what is going to happen, and if the operation is on the patient’s right shoulder, then the tube should rather not be in that side of the mouth.” *S3	Task management	The anaesthesiologist needs to incorporate knowledge about the operation into the planning of the anaesthesia.	Planning and preparing	Incorporates knowledge of the specific operation in the planning of anaesthesia
*“It is important to know the operation more or less, to know when it can be critical, and if there is bleeding and if it will be very painful afterwards.” *CA3				

NA: Nurse Anaesthetist; TA: Trainee Anaesthesiologist; CA: Consultant Anaesthesiologist; SN: Scrub Nurse; S: Surgeon

### Refinement of the ANTSdk prototype

The ANTSdk prototype was refined through discussions in two regional educational council meetings and included insights from parallel work on behavioural marker systems for surgeons, NOTSSdk,^15^ and nurse anaesthetists, N-ANTS.^16^ The final version of ANTSdk was translated to English by RMHGJ and back translated by a professional translator.

## Results

### Results from the group interviews

The group interviews lasted from 46 to 67 minutes. The ANTSdk prototype developed from the interviews encompassed four categories, 16 elements, and 69 behavioural markers. All of the quotations regarding NTS from the interviews fit within the four ANTS categories. Overarching themes from the interviews were: being aware of and communicating own abilities to the team (example behavioural marker: ‘introduces himself or herself to new team members and states his or her competencies’ and ‘appears calm’); working systematically (example behavioural marker: ‘summarises the situation for the team when needed; for example, using ABCDE systematics’ and ‘uses systematics in planning the task’); and speaking up to avoid adverse events (example behavioural marker: ‘says that a mistake is about to occur’ and ‘justifies when guidelines are not followed’). ‘Demonstrating self-awareness’ was included as a new element under the category ‘Situation Awareness’ and comprised behavioural markers such as, ‘recognises that personal feelings can make work difficult and acts accordingly’, ‘overestimates own competencies’, and ‘does not say whether (s)he is overloaded’. Multiple examples highlighted the consequence for team members and patients when anaesthesiologists lost or did not consider self-awareness during their actions. Table 2 provides examples of interview quotations that led to the formation of behavioural markers in all four categories.

### Results from the regional educational council meetings

After the first group of consultants and trainees discussed the ANTSdk prototype, 47 new behavioural markers were added. Further, 15 behavioural markers were added on the basis of the second group’s discussion. The participants stressed the anaesthesiologists’ leadership role in the OR; consequently, the ANTS category ‘Task Management’ was called ‘Leadership’. The participants emphasised that the ANTS element ‘Using authority and assertiveness’ was part of the leadership role and the element was moved from the ‘Team Working’ to the ‘Leadership’ category.

### The ANTSdk instrument

We developed short descriptions aimed at extracting the essence of the four categories (Table 3). The content was based on quotations from the interviews and discussions of the ANTSdk prototype. In total, 47% of the ANTSdk behavioural markers were found to be different from the ANTS behavioural markers. We used a five-point Likert scale (from ‘much below average’ to ‘much above average’) for the categories and elements, and a seven-point Likert scale (from ‘poor’ to ‘excellent’) to provide a global rating of NTS. The back-translation of ANTSdk did not reveal issues of understanding that needed clarification. Table 4 provides an example of the ANTSdk structure. The full version of ANTSdk including the rating form is available on the Internet.^20^

**Table 3.** Description of ANTSdk categories

Category	Definition
Situation awareness	Maintaining a dynamic attention to the situation by including information from the patient, the team, equipment, etc. and thinking ahead. Being aware of own abilities and continually assessing own handling of the situation.
Decision making	Assessing the situation and making a decision. Communicating plan and implementing decisions. Reassessing and adapting the strategy to the dynamic situation.
Team working	Facilitating collaboration through safe communication, coordinating tasks on the basis of assessment of team skills, ensuring that team members have the necessary understanding of the situation, and being aware of factors that can affect team members' ability to solve a problem.
Leadership	Organising and prioritising resources and activities to solve tasks on the basis of the department’s guidelines. Assuming a leading or non-leading role depending on the situation. Focusing on the safety and quality of the work.

**Table 4.** Overview of the ANTSdk categories, elements, and selected examples of behavioural markers

Categories	Elements	Examples of good behaviour	Examples of poor behaviour
Situation awareness	Gathering information	Focuses on the specific situation*	Does not use systematics when gathering information about the situation*
	Recognising and understanding contexts	Describes relevant changes in the patient’s status to the team and ensures that appropriate action is taken when needed	Does not point out relevant changes in a patient’s condition to the team*
	Anticipating and thinking ahead	Informs team members when a situation could develop critically	Refuses to respond to questions about alternative plans
	Demonstrating self-awareness	Knows own limits*	Exhibits inappropriate behaviour in relation to the situation*
Decision making	Identifying options	Summarises the situation for the team when needed; for example, using ABCDE systematics*	Does not consider differential diagnosis
	Choosing, communicating and implementing decisions	Uses the options available in the given situation*	Does not involve the team in decisions when relevant
	Reassessing decisions	Asks team members for input when reassessing*	Reassesses in too much detail and/or too often*
Team working	Exchanging information	Introduces her/himself to new team members and states competencies*	Gives too many orders at once*
	Assessing competencies	Reacts to signals from team members when they are losing focus and no longer can manage the task	Does not call for help if team competencies are insufficient
	Coordinating activities	Includes knowledge about team members' competences when tasks are distributed	Stays passive without participating in the coordination of activities
	Supporting others	Appears calm*	Seems unstructured and confused*
Leadership	Planning and preparing	Uses systematics in planning the task*	Does not make alternative plans *
	Prioritising	Adapts priority when changing conditions requires it*	Leaves the operating room when (s)he should be present*
	Identifying and utilising resources	Adapts task management to the team’s overall competencies*	Starts more activities than there are resources for*
	Using authority and assertiveness	Says if a mistake is about to occur*	Does not insist on working in quietness when needed*
	Providing and maintaining standards	Justifies when guidelines are not followed*	Fixates on using a single guideline although it does not fit the situation*

*Examples of some of the ANTSdk behavioural markers that are new compared with the ANTS behavioural markers.

## Discussion

This explorative study was conducted in two steps. First, OR team members were interviewed about anaesthesiologists’ NTS, data were analysed using qualitative directed content analysis, and the result was formulated into a ANTSdk prototype. Second, the ANTSdk prototype was discussed with anaesthesiologists from more than half the Danish anaesthesia departments, and the ANTSdk instrument was customised. The resulting ANTSdk instrument comprises four categories and 16 elements, each underpinned by numerous observable behavioural markers. We found an overlap between ANTS and ANTSdk on the higher levels (categories and elements). At the lower level (behavioural markers), approximately half of the behavioural markers differed between the instruments.

We called one category ‘Leadership’ instead of ‘Task Management’.

The Danish anaesthesiologist was described as stepping in and out of a leadership role shared with the surgeon; sometimes managing the smaller anaesthesia sub-team; and sometimes managing the overall situation for both the anaesthesia and the surgical sub-team.

The nurse anaesthetist maintaining the anaesthesia facilitates the latter function, enabling the anaesthesiologist to step back and gain an overview without being involved in solving specific technical tasks. Thus, the anaesthesiologist is able to exercise dynamic leadership that has been acknowledged as important for OR team safety performance and outcomes.^21^ Interview statements emphasized that the anaesthesia leadership was highly valued during emergencies, but was seldom needed during routine cases and could be problematic during intermediate cases for which the chain of command was less clear. Such a dynamic shift in leadership over time in response to the development of a case seemed an important aspect to train and anchor organisationally. Communication about the tasks in the OR between the different sub-teams was emphasised as being crucial for the benefit of the patient.

We included a new element, ‘Demonstrating self-awareness’ to gather the behavioural markers regarding this issue because the ability to exercise self-awareness was regarded as important in the interviews. The new element is similar to a new element, ‘Monitoring own performance’, which was included during the customisation of NOTSS to the Danish setting as NOTSSdk.^15^ The changes in ANTSdk and NOTSSdk might be caused by a Scandinavian cultural influence. Denmark is considered a feminine society by Hofstede, in which decision making achieved through involvement and equality is considered important as opposed to, in Hofstede’s findings, a more masculine and individualistic country such as the United Kingdom.^22^ Consequently, carefully adjusting how one presents oneself in relation to other OR members in Denmark is highly valued. This phenomenon is in line with the changes found in NOTSSdk and N-ANTS.^15^^,^^16^ The inclusion of the element ‘Demonstrating self-awareness’ is also in line with research on the importance of a timely transition between the automatic and the effortful mode during challenging tasks and through the essential skill of self-monitoring.^23^^,^^24^

The focus on team issues in ANTSdk might be partly explained by the multi-professional and multi-disciplinary data sampling strategy, including interviewing members of all staff groups of the OR team to ensure a broad perspective on anaesthesiologists’ NTS. Exemplified under the ANTSdk category ‘Team Working’, in which the importance of stating one’s competences when introducing oneself to other team members is highlighted in interview quotations and the derived behavioural marker (Table 2). Involving other professions in the data collection likely involves more interactional and teamwork aspects, as our research group previously found.^15^^,^^16^ A part of the explanation for the results in the Danish setting is likely the close teamwork with nurse anaesthetists, which requires good communication skills. A focus on patient safety in the OR, such as the WHO checklist, also highlighted the importance of teamwork.^25^ The interview participants stated that all members of the Danish OR team could likely demand justification and information on actions and further plans from the anaesthesiologist, which might reflect the concept that hierarchy and power distance are less prominent in Denmark than in the United Kingdom.^22^ In the ANTSdk ‘Decision Making’ category, the need for justification and communication of decisions are emphasised (Table 2).

ANTSdk highlights the need for the current physicians’ ability to reflect on their own skills compared with ANTS. This change might have been facilitated by the increasing focus on the CanMEDS roles for physicians during the last decade.^26^ The acknowledgement that physicians need to be not only technical and medical experts but should also possess other skills is emerging throughout healthcare. The influence of implementing the CanMEDS framework for in-training assessment for formative feedback in medical specialist training in Denmark might have had an effect on the expectations of the anaesthesiologist to act reflectively.

Overall, the major changes between ANTSdk and ANTS that were primarily found in the behavioural makers might be explained by different tasks, responsibilities, and cultures. Similar findings have been reported from the customisation of NOTSSdk from NOTSS and adaptation of N-ANTS from ANTS.^15^^,^^16^

These findings may also be interpreted through the lens of Activity Theory.^17^ The categories together describe the non-technical part of the ‘activity’ of providing anaesthesia. The elements and behavioural markers then correspond to the goal-oriented ’actions’ in increasing detail and with increasing sensitivity to context. Over time and with experience, parts of the actions might become automatic and are then described as so-called ‘operations’ in the terminology of Activity Theory.^17^ This change corresponds to our assumption that the goal-guided activity of providing anaesthesia is similar across contexts but that differences appear as the behaviours become more concrete and context dependent. In addition to the technical aspects of any task, developing an awareness of the situation is necessary for making decisions about what to do and to organise task execution among the different people involved. Building situation awareness requires similar steps in all different areas: gathering information, understanding it, and predicting the future.^27^ Decision making likely always involves an understanding and an assessment of different options. However, the manner in which these elements are put into concrete and context-dependent practice requires very specific steps. Whereas an anaesthesiologist in one country might make a decision alone, in another country (s)he might first discuss the situation with colleagues before making a decision. We interpret this difference as an indication that NTS might be generic across disciplines and countries at a higher level.

### Discussion of methods used

We considered whether using the existing ANTS categories limited the research process by locking the research team into a rigid pre-understanding. However, taking into account the comprehensive scientific work behind the development of ANTS,^2^ we consider this approach to be a strength of ANTSdk. We chose a qualitative research approach because it is recognised as an important methodology when exploring how anaesthesiologists think.^28^ The recruitment of participants through their local leaders might have resulted in a selected sample of persons interested in the topic. Although introducing a potential bias, we considered the best strategy encountering these individuals because they could assist in identifying whether and how the original ANTS did (or did not) fit the Danish organisation and culture. We performed the interviews mono-professionally to minimise possible bias from interactions across disciplines. This approach may have reduced a potentially positive effect of having OR team members from different professional groups reflect together. To minimise reporting bias from anaesthesiologist trainees who might feel pressure from the consultants, we interviewed trainees separately.

The semi-structured group interview technique with open questions was used as a way to gain an understanding of participants’ perspectives on specific situations and experiences expressed in their own words.^28^ Using their own words was crucial because participants’ statements about anaesthesiologists’ good and poor behaviour were used to define the content of ANTSdk. We chose group interviews to allow participants to inspire each other, acknowledging that some personal views might be held back.^29^ The same order of the questions in the interview guide was used for all of the interviews. Consequently, less time was spent discussing the ANTS categories ‘Situation Awareness’ and ‘Decision Making’ because they were discussed after ’Team Working’ and ‘Task Management’. This exclusion may potentially have biased the results; however, the semi-structured nature of the interviews enabled us to frequently touch on topics regarding these categories when discussing other categories. The generalizability of the results might be compromised because we performed the interviews in one university hospital. However, interviewing representatives from all members of the OR team, including five different surgical specialities, ensured a large variety of views. Further, the two groups of anaesthesiologists discussing the ANTSdk prototype in the regional education council meetings comprised a broad span of university hospitals and experience, from inexperienced trainees to very experienced consultants with masters’ degrees in medical education from two out of three Danish educational regions. The results might be less transferable to non-university hospitals. We chose a five-point Likert scale to rate the observed skills in the categories and elements to allow raters to differentiate behaviour and to avoid the potential ceiling effect previously found with the four-point Likert scale used in ANTS.^30^ We added a global rating scale to ensure that the raters assessed the overall NTS and used the scale during evaluation studies of ANTSdk to ensure that elements and categories were complete in rating NTS. A seven-point Likert scale was used for the global rating to enhance rater reflections on overall performance and to avoid averaging of ratings from the categories and elements. We kept space on the rating form for free-text notes to enhance the value of the feedback given by the rater. This feedback is important because a numeric evaluation holds little information in itself. The combination of numeric and narrative feedback will provide the rated anaesthesiologist with specific feedback on observed behaviour together with a numeric trend during a training period. This combination of assessment types is increasingly being described as a useful feedback method.^31^

### Implications for medical education

ANTSdk provides common terminology for use in medical education and the words for actions that most clinicians use to some degree without having the comparable terms to describe these actions. In this sense, ANTSdk can fulfil an educational function by raising awareness for the terms and facilitating their discussion. ANTSdk facilitates clarification on what the CanMEDS roles encompass as four of the seven roles are directly applicable: Collaborator, Communicator, Manager, and Professional.^26^ ANTSdk potentially helps describe the goals related to each of these roles in more depth on the basis of the behavioural markers to structure learning and assessment.

## Conclusion

ANTSdk was customised using the identified non-technical skills for anaesthesiologists and ANTS as a basis. The ANTS category ‘Task Management’ was named ‘Leadership’. A new element, ‘Demonstrating self-awareness’ was added. Differences between the two instruments are mainly apparent in the behavioural markers. ANTSdk comprises four categories and 16 underpinning elements supported by numerous examples of good and poor behaviour. Danish anaesthesiologists found ANTSdk to be usable. Identifying NTS through semi-structured group interviews and analysing them through direct content analysis proved useful to customise an assessment instrument for another setting.

## 

### Conflict of Interest

The authors declare that they have no conflict of interest.
